# Electric Field Induced Fluorescence Modulation of Single Molecules in PMMA Based on Electron Transfer

**DOI:** 10.3390/ijms130911130

**Published:** 2012-09-06

**Authors:** Ruiyun Chen, Yan Gao, Guofeng Zhang, Ruixiang Wu, Liantuan Xiao, Suotang Jia

**Affiliations:** State Key Laboratory of Quantum Optics and Quantum Optics Devices, Laser Spectroscopy Laboratory, Shanxi University, Taiyuan 030006, China; E-Mails: chenry421@163.com (R.C.); ggnnool@163.com (Y.G.); gfzhang@mail.sxu.cn (G.Z.); Wurx464628021@163.com (R.W.); tjia@sxu.edu.cn (S.J.)

**Keywords:** single-molecule fluorescence, modulation, external electric fields, polarization

## Abstract

We present a method to modulate the fluorescence of non-polar single squaraine-derived rotaxanes molecules embedded in a polar poly(methyl methacrylate) (PMMA) matrix under an external electric field. The electron transfer between single molecules and the electron acceptors in a PMMA matrix contributes to the diverse responses of fluorescence intensities to the electric field. The observed instantaneous and non-instantaneous electric field dependence of single-molecule fluorescence reflects the redistribution of electron acceptors in PMMA induced by electronic polarization and orientation polarization of polar polymer chains in an electric field.

## 1. Introduction

The desire to exploit single-molecule spectroscopy [1,2] to optical nonlinearity [3], electron-optical devices [4], and quantum optics [5] has triggered a large number of studies on manipulating single molecule fluorescence (SMF). To date, there are two main schemes used to manipulate SMF. On the one hand, SMF is controlled by using metal tips or gold nanoparticles as nanoantenna [6–9]. It is found that the local field enhancement leads to increased excitation rates, whereas non-radiative energy transfer to the nanoantenna leads to a decrease of quantum yield of single molecules. On the other hand, an electric field (EF) is applied on the film electrodes of multilayer devices where single molecules are doped in a thin polymer film [10–12]. Numerous studies on interfacial electron transfer dynamics [13–17] between single molecules and semiconductor electrodes have proposed that electron transfer has a strong impact on the SMF. Observation of EF dependence of SMF has shown that the local environment of the polymer has a great effect on the response of the fluorescence emission to the EF. It is suggested that accepting (or trapping) states in polymers plays an important role in the fluorescence modulation of single molecules. The charge accepting states are inherent to disordered materials and proposed to be a general source of fluorescence intermittency of single emitters such as quantum dots [18–20] and dye molecules [21,22]. A trap model proposes that the charge is assumed to tunnel back and forth between the emitter and immobile trap sites in the surrounding dielectric environment [23].

PMMA is a polar polymer with a dielectric constant of 3.4 [20], which is widely used in the design of single photon sources [24] and quantum devices [25] based on single molecules or nanocrystals. PMMA has a polar ester group –COOCH_3_ with a dipole moment of 1.6 Debye [26]. In the studies of EF-induced fluorescence modulation of single conjugated polymer chains in a PMMA matrix, it was found that the modulation directly is attributed to EF-induced long-living on-chain hole polarons in the single conjugated polymer [10,12]. However, limited experiments provide information on the effect of EF-induced variation of microscopic nature of inhomogeneous PMMA matrix on the fluorescence of single molecules. In contact charging or contact electrification experiments at metal-polymer or metal-insulator interfaces, charge flow from the metal into the PMMA is observed [27–30]. It is suggested that the charges in PMMA are localized due to a relaxation of the surrounding polymer matrix. Fluorescence intermittency has been studied in single dye molecules [22] and nanocrystals [20] in PMMA matrix. It is found that the intermittency of single emitter responds to the polarity of the surrounding polar PMMA polymer [20] and the carbonyls in the PMMA ester groups serve as electron acceptor sites [10]. Owing to the polar nature of PMMA, the redistribution of acceptor states seems to responds to the polarization of the surrounding matrix under external EF, which provides a route to control the fluorescence of single molecules in PMMA by applying an external EF.

In this paper, fluorescence of non-polar single squaraine-derived rotaxane (SR) molecules in a polar PMMA matrix is investigated under the influence of an external EF. Owing to the symmetrical structure of non-polar SR molecules, the effect of external EF on the polarization of single molecules can be neglected compared to the polar PMMA polymer chains. Therefore, the manipulation of SMF would only be attributed to the EF-induced variation of the microscopic environment of a PMMA matrix.

## 2. Results

### 2.1. Diverse Responses of SMF in PMMA under EF

**Figure 1:**
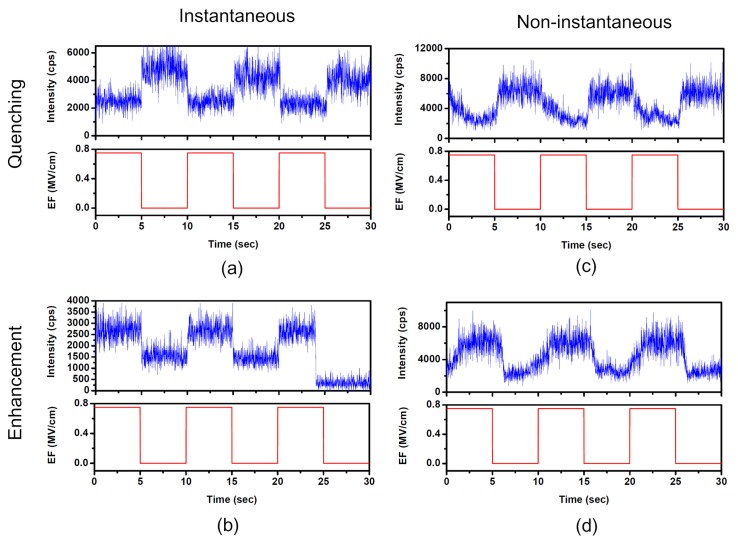
Diverse fluorescence response of single squaraine-derived rotaxane (SR) molecules in PMMA matrix as a function of time at an electric field (EF) of 0.75 MV/cm.

A square alternating voltage is applied to two neighboring arms of the electrodes where the single molecules are dispersed, which yields an EF of 0.75 MV/cm. We measured the fluorescence time traces of 147 single molecules, with sufficient observation time and adequate signal-to-noise ratio. Figure 1a,c shows that fluorescence of some single molecules is quenched when EF is applied. The probability of EF-induced fluorescence quenching is approximately 31%. Figure 1b,d shows that fluorescence of some molecules is enhanced when EF is applied. The probability of EF-induced fluorescence enhancement is nearly 26%. However, about 43% of the molecules measured did not show obvious enhancement or quenching when EF was applied (not shown).

When an external EF is applied to the sample, some molecules whose fluorescence is modulated show an instantaneous fluorescence response while others show a non-instantaneous response. Figure 1c,d shows the typical fluorescence intensity time traces of two single molecules with non-instantaneous response compared with instantaneous response, which is shown in Figure 1a,b. Figure 1c shows that the fluorescence of such a molecule is quenched with a time constant of 1.23 s when EF is turned on and recovered with a time constant of 0.32 s when EF is off. Both the quenching and recovery traces are exponentially fitted. Figure 1d shows that the fluorescence of such molecules is enhanced with a time constant of 1.31 s when EF is on and recovered with a time constant of 0.35 s when EF is off.

### 2.2. Diverse Modulation Depth and Response Time of SMF in PMMA under EF

**Figure 2:**
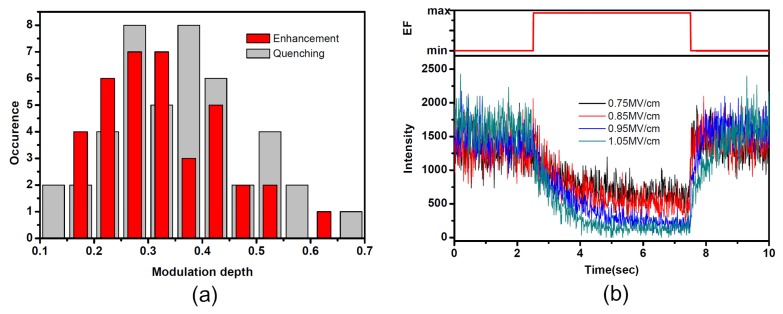
(**a**) Distribution of EF-induced fluorescence modulation depth M for fluorescence quenching (light gray, 46 molecules) as well as fluorescence enhancement of single SR molecules (red, 38 molecules) in PMMA matrix at 0.75 MV/cm; (**b**) Typical SMF modulation pattern obtained under different EF.

In order to investigate the heterogeneity of PMMA matrix under EF, modulation depth (M) was used to qualitatively describe the response of SMF under EF, which is defined as (*I*_max_ − *I*_min_)/*I*_max_, with *I*_max_ and *I*_min_ being the maximum and minimum fluorescence intensity of the individual molecule. Figure 2a shows the distribution of the EF-induced fluorescence modulation depth of 84 single SR molecules at an EF of 0.75 MV/cm. While 38 of these molecules showed fluorescence enhancement, the others showed fluorescence quenching. The most probable value of modulation depth is about 0.32 both for enhancement and quenching at 0.75 MV/cm.

Figure 2b shows the modulation effect of a single molecule, of which the fluorescence was quenched under EF. The single molecule survived for such a long time that fluorescence time traces under EF intensity of 0.75, 0.85, 0.95 and 1.05 MV/cm were obtained. The modulation depth under different EF was 0.53, 0.67, 0.84 and 0.88, respectively. It was found that the bigger the EF intensity, the larger the modulation depth. The EF-induced fluorescence-quenching time and recovery time traces were exponentially fitted. Under an EF of 0.75, 0.85, 0.95 and 1.05 MV/cm, the fluorescence quenching times were 1.29, 1.27, 1.04 and 0.68 s, while the fluorescence recovery times were 0.23, 0.28, 0.42 and 0.63 s, respectively. It was found that under larger EF the field induced response was faster and the recovery was slower.

**Figure 3:**
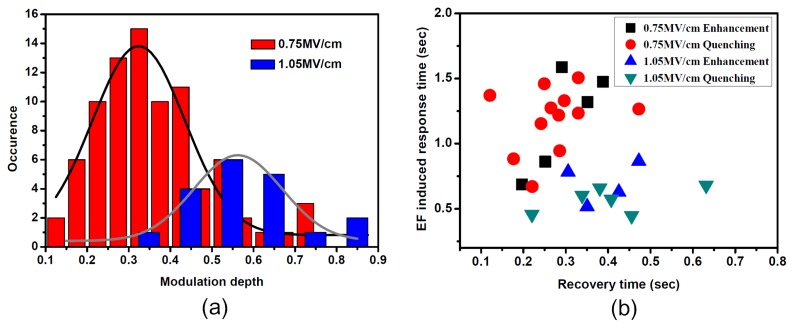
(**a**) Distribution of EF-induced fluorescence modulation depth for single SR molecules in PMMA at EFs of 0.75 MV/cm and 1.05 MV/cm, respectively; (**b**) Distribution of the transient response for field-induced fluorescence quenching molecules and field-induced fluorescence enhancement molecules under EFs of 0.75 MV/cm and 1.05 MV/cm, with the vertical axis being the EF-induced response time of fluorescence and the horizontal axis the recovery time of fluorescence when the EF is turned off.

In addition to the qualitative variation of the fluorescence of individual molecules under different EFs, the statistical distribution of modulation depth and transient response time of single SR molecules under EFs of 0.75 MV/cm and 1.05 MV/cm were investigated. Figure 3a shows the distribution of fluorescence modulation depth of the total response molecules at an EF of 0.75 MV/cm and 1.05 MV/cm. It was found that the modulation depth became larger at higher field amplitudes. The most probable value of modulation depth was moved to 0.55 at an EF of 1.05 MV/cm compared with 0.32 of 0.75 MV/cm. Figure 3b shows the time constants of molecules, which have non-instantaneous fluorescence response under EF of 0.75 MV/cm and 1.05 MV/cm. Under an EF of 0.75 MV/cm, it was found that the time constants of EF-induced fluorescence quenching or enhancement were larger than that of fluorescence recovery after the EF was turned off. Under the EF of 1.05 MV/cm, the average time constant of EF-induced quenching or enhancement was found to be smaller than the value at 0.75 MV/cm. Meanwhile, the average time constant of fluorescence recovery at EF of 1.05 MV/cm seemed to be similar to that of 0.75 MV/cm.

## 3. Discussion

**Figure 4:**
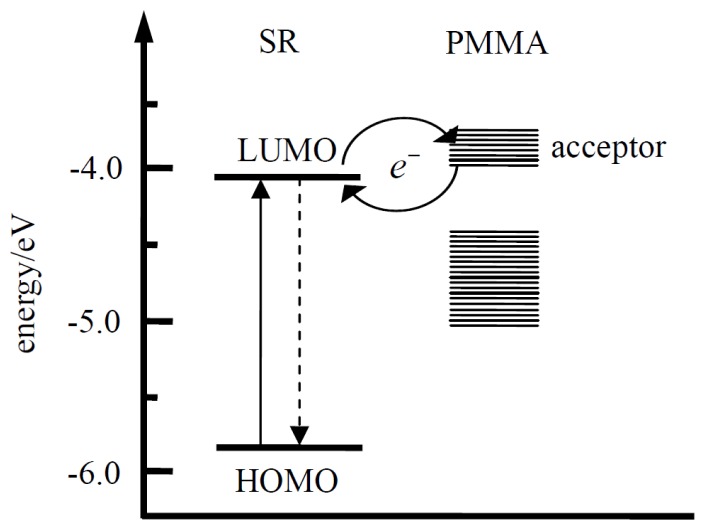
HOMO/LUMO energy level scheme of SR molecule (adapted from [31]) as well as electron acceptor states in PMMA (adapted from [32]) with respect to the vacuum level.

After a single molecule is photoexcited, the resulting first excited state may relax via several competing pathways: radiative or non-radiative decay to the ground state, formation of a long-living triplet state or charge separation. It has been proposed in numerous studies that charge transfer is a dominant fluorescence-quenching pathway, which could be controlled by external EF. Therefore, the fluorescence efficiency may be reduced or enhanced by EF-induced modulation of the charge transfer rate. The modulation effect in Figure 1 can be properly explained by diminishment or enhancement of the fluorescence-quenching pathway induced by external EF. It has been proposed that the carbonyls in the PMMA ester groups can serve as electron acceptors surrounding single molecules [10]. The acceptors are well known to play an important role in polymers in contact charging or contact electrification experiments where a charge flow from the metal into the polymer was observed. Figure 4 shows the HOMO/LUMO levels of an SR molecule adapted from ref. [31] and the energies of electron acceptor states in PMMA determined by contact charging experiments [32]. Based on the energetic considerations, forward and backward electron transfer may happen between single molecules and acceptors in the surrounding environment. Excited electrons of single molecules may forward electron transfer to acceptors in the surrounding environment, which will lead to the decrease of the fluorescence quantum yield. Meanwhile, the electrons trapped in acceptors would also back transfer to the single molecule to renew excitation and emission cycles.

If the coupling between single molecules and surrounding acceptors is enhanced owing to the external EF, the electrons are more easily trapped by the acceptor sites, which results in the decrease of the SMF quantum yield. The forward transfer rate is increased and the backward transfer rate is decreased. The fluorescence of single molecules is quenched, as shown in Figure 1a,c. However, if the coupling between single molecule and surrounding acceptor sites is suppressed, the forward transfer rate is decreased. The fluorescence quenching effect is diminished and the fluorescence quantum yield of single molecules is enhanced, which leads to the enhancement of SMF as shown in Figure 1b,d. For the single molecules, which had no response to the external EF, there is no such coupling between single molecule and electron acceptors. Owing to the heterogeneity of local environment of single molecules, the coupling interaction between an individual molecule and its environments would be different from molecule to molecule when EF is applied, which results in the distribution of modulation depth in Figure 2a.

In order to explain the instantaneous and non-instantaneous response of single molecule fluorescence under external EF, the field-induced polarization of PMMA polymer chains is proposed. As an amorphous polar polymer, PMMA has numerous dipole points in random directions. When an external EF is applied, two types of responses exist: First, the forces on the electrons will induce an extra dipole moment on polymer chains, which is called electronic polarization. The electronic polarization processes is ultrafast (about a femtosecond) [33]. Second, the external EF tends to line up the permanent dipoles of polymer chains to the direction of EF, which is called orientation polarization. Both electronic polarization and orientation polarization would induce the redistribution of electron acceptors in PMMA. Owing to the ultrafast electronic polarization of polymers, electron acceptors will have a quick redistribution under EF, which induces the instantaneous response of SMF with respect to the external EF. For the non-instantaneous response of SMF under EF, the situation is more complicated. In addition to the ultrafast electronic polarization, orientation polarization exists in some polar polymer chains under EF. However, owing to the viscosity of the polymer, it takes a certain amount of time for the polar chains to turn toward the direction of the EF, which will result in a slow orientation polarization and hence a slow redistribution of electron acceptors surrounding the impurity single molecules. Thus the forward transfer rate and backward transfer rate slowly reach an equilibrium state and the fluorescence of these single molecules shows non-instantaneous response with respect to the external EF.

The results in Figures 2 and 3 are strong evidence that the EF-induced polarization of PMMA polymers contribute to the redistribution of electron acceptors in the dielectric matrix, thus leading to the modulation effect of SMF when EF is applied. At larger EF, both electronic polarization and orientation polarization are much more easily induced. Larger redistribution of electron acceptors may cause larger fluorescence modulation depth in individual molecules, which can be seen in Figures 2b and 3a. Meanwhile, it is found in Figure 3b that the EF-induced response time of SMF under larger EF is found to be shorter than that under smaller EF. It is reasonable to think that the line-up of polymer chains toward the direction of EF is easier under larger EF than smaller EF. However, when EF is turned off, the slow relaxation of fluorescence is only attributed to the viscosity of the polymer, which yields a similar recovery time under larger EF and smaller EF.

## 4. Experimental Section

### 4.1. Sample Preparation

**Figure 5:**
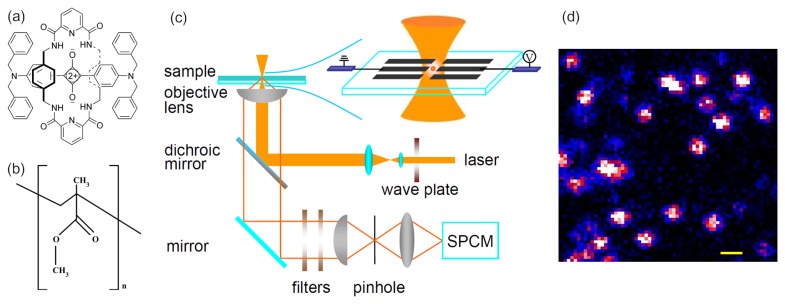
(**a**) and (**b**) are the chemical structures of squaraine-derived rotaxanes (SR) dye molecule and PMMA polymer chain, respectively; (**c**) Schematic of experimental setup for single molecule detection based on a confocal microscope. The enlarged part shows the pattern of electrodes; (**d**) Shows the confocal scanned fluorescence image (10 × 10 μm^2^) of SR molecules sandwiched PMMA film.

Squaraine-derived rotaxane dye molecules [31], whose maximum excitation wavelength is 650 nm and maximum fluorescence emission wavelength is 678 nm (1.82 eV), was purchased from Molecular Targeting Technologies Inc. (West Chester, PA, USA) The chemical structure of SR is shown in Figure 5a. PMMA (MW = 15,000, *T*_g_ = 82 °C) was purchased from Aldrich. The chemical structure of the PMMA chain is shown in Figure 5b. The PMMA-toluene co-solution (20 g L^−1^ PMMA) was first spin-coated at 3000 rpm onto a glass cover slip on which aluminum electrodes were fabricated. The aluminum electrodes with a thickness of 1.5 μm and a gap of 10 μm between two adjacent arms were prepared using aluminum alloy e-beam evaporator and standard optical lithography. The sample was prepared by spin-coating 20 μL water solution containing SR molecules at a concentration of 10^−10^–10^−9^ M into the electrode gaps. A PMMA film was further spin-coated onto the sample. The sandwich-like sample was carefully prepared to isolate the molecules from the electrodes to avoid charge injection and to prevent oxygen-induced fast photobleaching. The sample was heated under vacuum to 373 K for 3 h then held overnight under vacuum at room temperature to remove the remaining solvent and relax stresses induced by the spin-coating process. The resulting thickness of the sample films amounts to about 200 nm. The upper right image in Figure 5c shows the pattern of electrodes. Square alternating voltage was applied to two neighboring arms of aluminum electrodes at a frequency of 0.1 Hz, which yielded a parallel EF between the two neighboring arms of electrodes. The single molecules were dispersed in the electrode gaps with random directions with respect to the parallel EF.

### 4.2. Fluorescence Imaging of Single Dye Molecules in PMMA

Single molecule images were taken with a home-built sample scanning confocal microscope based on an inverted microscope (Nikon ECLIPSE TE2000-U). A 70-picosecond-pulse diode laser (*λ* = 635 nm, repetition rate 40 MHz) was used to excite the samples. The excitation light was, after circularly polarized by a 1/4-wave plate, directed by a dichroic mirror (BrightLine, Semrock, Di01-R635-25×36, Tampa, FL, USA) towards an oil immersion objective (Nikon, 100×, 1.3NA) to obtain a diffraction limited spot on the sample plane. Fluorescence light of single molecule was collected by the same objective and passed through the dichroic mirror, an emission filter (BrightLine, Semrock, FF01-642/LP-25-D), and a notch filter (BrightLine, Semrock, NF03-633E-25) to block the backscattered laser light in fluorescence. The fluorescence light is then focused onto a 100 μm spatial filter and collected by an avalanche photodetector (PerkinElmer, SPCM-AQR-15, Wiesbaden, Germany). Confocal fluorescence images were acquired by raster scanning the sample in the focal plane of the laser beam and recording the fluorescence for each image pixel. Figure 5d shows a typical image of single SR dye molecules within 10 μm × 10 μm area. The image is recorded in 225 s (100 pixels × 100 pixels). The integration time of each pixel was set to 10 ms. Single molecules were excited with a laser intensity of 3.6 kW/cm^2^ at 40 MHz. The concentration of SR molecules was kept at such a low level that either one molecule or no molecule was in focus. The bright features in Figure 5d represent the fluorescence from individual molecules.

## 5. Conclusions

In conclusion, EF-induced fluorescence modulation was found in individual SR dye molecules dispersed in PMMA matrix. Both EF-induced fluorescence quenching and enhancement are found. It is found that EF-induced redistribution of electron acceptors in PMMA contributes to the diverse modulation of SMF. Instantaneous and non-instantaneous responses of SMF are found in the modulation. It is proposed that orientation polarization of polar polymer chains, which is an intrinsic property of polar polymers in EF, induces the non-instantaneous response of SMF compared with the instantaneous response due to the ultrafast redistribution of electron acceptors induced by electronic polarization. Distribution of modulation depth shows the inhomogeneous microscopic nature of PMMA matrix. For the single molecules, which undergo non-instantaneous fluorescence response, a faster response time is found when larger EF is applied, which can be explained in terms of faster redistribution of electron acceptors due to orientation polarization. The results could open up a new path to investigate the microscopic dielectric properties of polymers used in organic optical-electronic devices.
